# Increased Use and Large Variation in Strong Opioids and Metamizole (Dipyrone) for Minor and Major Musculoskeletal Injuries Between 2008 and 2018: An Analysis of a Representative Sample of Swiss Workers

**DOI:** 10.1007/s10926-023-10115-5

**Published:** 2023-04-11

**Authors:** Dominic Müller, Stefan M. Scholz, Nicolas Fabrice Thalmann, Maurizio Alen Trippolini, Maria M. Wertli

**Affiliations:** 1 Department of General Internal Medicine, University Hospital of Bern, Inselspital, Freiburgstrasse 18, 3010 Bern, Switzerland; 2 Department of Statistics, Suva (Swiss National Accident Insurance Fund), Lucerne, Switzerland; 3 Bern University of Applied Sciences, School of Health Professions, Murtenstrasse 10, 3008 Bern, Switzerland; 4 Institute of Physiotherapy, University Hospital of Bern, Inselspital, Freiburgstrasse 18, 3010 Bern, Switzerland; 5 Evidence-Based Insurance Medicine (EbIM), Division of Clinical Research, Department of Clinical Epidemiology, University Hospital Basel, University of Basel, Totengässlein 3, 4051 Basel, Switzerland; 6 Department of Internal Medicine, Kantonsspital Baden, Im Ergel 1, 5404 Baden, Switzerland

**Keywords:** Analgesic, Opioid, Pain, Injuries, Switzerland, Musculoskeletal injuries, Pain medication, Non-opioid, Workers compensation

## Abstract

**Purpose:**

Musculoskeletal (MSK) injuries are a major contributing factor for chronic pain. To date, little is known how pain medication use in MSK injuries has changed over time. We assessed pain medication prescription for MSK injuries in a representative sample of Swiss workers between 2008 and 2018.

**Methods:**

Retrospective analysis of the Swiss Accident Insurance Fund (Suva) data. We calculated annual pain medication use, treatment days, and costs associated with pain medication use in minor and major MSK injuries.

**Results:**

In total, 1,921,382 cases with MSK injuries with ≥ 1 pain medication were analyzed. Whereas MSK injuries with ≥ 1 pain medication increased by 9.4%, we observed a larger increase in metamizole (+ 254%), strong opioids (+ 88.4%), coxibs (+ 85.8%), and paracetamol (+ 28.1%). Strong opioids were increasingly used in minor (+ 91.4%) and major (+ 88.3%) injuries. The increase in metamizole (+ 390.6%) and coxibs (+ 115.5%) was larger in minor injuries compared to major injuries (+ 238.7% and + 80.6%, respectively). Medical expenses decreased in all medications except for strong opioids where a substantial increase was observed (+ 192.4% in minor; + 34% in major injuries).

**Conclusions:**

We observed a disproportionate increase in metamizole, strong opioids, coxibs, and paracetamol prescriptions even in minor MSK injuries between 2008 and 2018. Whereas treatment costs decreased for all pain medications, there was a substantial increase in strong opioids. A more liberal prescription practice of opioids conflict with current evidence-based practice recommendations and need to be addressed by physicians and policy makers.

**Supplementary Information:**

The online version contains supplementary material available at 10.1007/s10926-023-10115-5.

## Background

Chronic musculoskeletal (MSK) pain is among the leading causes for pain related disability and MSK diseases account for 16% of years lived with disability [1]. MSK injuries are a major contributing factor for chronic pain [2–5]. Pain due to MSK injury after a road traffic accident persists between 30 to 54% at 6 months [5] and beyond 1 year in 22% of the patients, respectively [6]. According to the Swiss [7] and the Australian [8] workers compensation statistics, MSK injuries accounted for 55% to 63% of work-related accidents and 82% of non-occupational accidents in 2018 [7, 8]. Although the mean number of sick leave days was moderate (11 days compared to 32 days after a fracture) [9], the high number of cases result in a significant burden to the health care system.

After an injury, acute pain management includes the use of pain medications [10–12]. Guidelines recommend non-opioid analgesic combined with non-pharmacological treatments as the first choice, followed by weak opioids and strong opioids for moderate to severe pain [11, 13]. In MSK pain, opioids have been shown to be no more effective than non-opioid pain medications but were associated with adverse effects [14–18]. In particular of concern are cognitive effects (e.g. drowsiness, cognitive impairment), nausea, hyperalgesia, the risk of opioid abuse or dependence [19–22], risk of overdose in high doses [23], emergency department visits, hospitalizations, and death [24]. Further, long-term opioid use in chronic MSK pain resulted in a poorer quality of life without improvement in function or pain control [19, 25]. Opioid dose reduction or discontinuation may lead to a reduction of pain severity, improved function, and life quality [26]. Therefore, opioids should be used with caution due to side effects and the very small effect on pain and function [18, 27] and be limited to cases of severe injury or intolerance of first-line therapy [10–12, 18, 27].

Despite the guideline recommendations, opioids are increasingly used in non-cancer related pain [28]. Globally, opioid use doubled between 2001–2003 and 2011–2013 mainly in North America (2.2-fold increase), Western and Central Europe (3.0-fold increase), and Oceania (4.0-fold increase) [28]. Increase in opioid use is often based on consumer data [29] or insurance data [30, 31] without clinical information. For example, in a study which analyzed claims data from a single health insurer, the use of strong opioids increased by 121% between 2008 and 2013 in Switzerland [31]. However, it remains unclear whether this increase was mainly due to more opioid use in severe diseases and at the end of life due to improved palliative care. In addition, it’s unknown whether pain medication use in workers with MSK injuries in Switzerland has changed over the last decades and whether changes differed between distinct pain medication groups.

Understanding the changes over time in prescription practices in MSK injuries may shed more insight. Therefore, the aim of this study was to describe changes in pain medication prescriptions in well-defined patient population. We analyzed all MSK injuries in a representative sample of Swiss workers between 2008 and 2018. We hypothesized that strong opioids are increasingly prescribed in minor MSK injuries which may indicate a wider use of opioids in situations where non-opioids are the preferred choice.

## Methods

### Study Design

Retrospective insurance claims analysis. The study was conducted following the International Society for Pharmacoeconomics and Outcomes Research (ISPOR) checklist for retrospective database studies [32].

### Data Sources

We used insurance claims data from the Swiss National Accident Insurance Fund (Suva) database. In Switzerland, all employees and all unemployed persons are covered by a compulsory accidence insurance according to the Swiss Accident Insurance Act. This insurance covers costs (wage compensations during work incapacities, long-term disability pensions, medical treatment costs and other medical expenses) of occupational and non-occupational accidents as well as for occupational diseases. Suva is the largest accident insurer in the country and insures mainly workers in the labor industries, and unemployed job-seeking persons. With approximately two million insured people from all cantons of Switzerland (corresponding to half of the Swiss active workforce) the data is highly representative [33].

Administrative data from the injury claims forms were used as source for sociodemographic information (sex, age at the date of the accident, and canton of residence), injured body part, type of injury, and circumstances of the accident (during work or during leisure time). In patients with more than one accident during the study period, each claim was included as a separate injury.

Data on healthcare expenses for pain medication were retrieved from the administrative Suva database on healthcare costs. The Suva database is fed directly from the electronic billing systems and all costs are attributed to a related case. It comprises data by granularity of invoice line items, with either pharmacode or Global Trade Item Number (GTIN) code, descriptive text, date, quantities, and invoiced amount of the line item. Pharmacode, GTIN code, and descriptive text were used to identify pain medication.

### Study Population

We included all MSK injury claims registered in 2008 to 2018. MSK injuries were identified by injury codes and the affected body parts. Included were MSK fractures, sprain (dislocation, sprain, and strain), rupture (rupture and tear), contusion (contusion and bruises), superficial (superficial injuries and cuts), and other MSK injuries (bites, foreign bodies, inflammation, edema, and bullet wound). We excluded claims for amputations, burns, poisons and chemical burns, injuries of the respiratory and internal organs, and claims for loss of sexual organs / reproductive ability. We also excluded claims for injuries that resulted in tetra- or paraplegia, claims for mental and physic shocks (allergic, hypothermia, heatstroke), and simple injuries of teeth, eye, ear, superficial abrasions, and fatalities. Finally, we excluded injuries where the injured body part or type of injury was unknown and cases with claims from outside of Switzerland.

### Follow-Up Duration

After registration of an accident (referred to hereafter as a “claim”), each claim was followed-up for 2 years (730 days). In case of several accidents, each claim was separately followed up for 730 days. Medication costs are not available for in-patients during their hospital stay, because for in-patients` diagnosis related group (DRG) flat rates apply in Switzerland, which include medication.

### Operational Definitions

#### Injury Severity

Accidents were divided into minor cases (less than 3 days absence from work) and major cases with daily allowances (which are paid when absence from work is more than 3 days).

#### Pain Medication Prescription

Pain medication prescription was assessed during the first 730 days after the date of the injury by identifying the appropriate WHO ATC codes. The WHO ATC/DDD system allows standardization of drug groupings and a stable drug utilization metric to enable comparisons of drug use between countries [34]. The defined daily dose (DDD) is provided by the WHO ATC and is based on the assumed average maintenance dose per day for a drug used for its main indication in adults [34]. Non-opioid pain medications included: paracetamol (ATC codes N02BE01, N02BE51), non-steroidal anti-inflammatory drugs (NSAIDs, M01AA, M01AB, M01AC, M01AE, M01AG, M01AX), coxibs (COX-2-inhibitors, M01AH), and metamizole (N02BB02, N02BB52). Weak opioids (defined as opioid formulations with a morphine conversion factor of ≤ 0.3) included dihydrocodeine (N02AA08), codeine (N02AA59, N02AJ06), tilidine (N02AX01), tramadol (N02AX02, N02AX52, N02AJ13), and tapentadol (N02AX06). Strong opioids (defined as all other opioids) included morphine (N02AA01), hydromorphone (N02AA03), nicomorphine (N02AA04), oxycodone (N02AA05, 02AA55), pethidine (N02AB02), fentanyl (N02AB03), buprenorphine (N02AE01), nalbuphine (N02AF02), buprenorphine (N07BC01), and methadone (N07BC02). Opioids only used within a drug substitution program (i.e., diamorphine N07BC06 Diaphin®) were excluded from the analysis.

#### Pain Medication Dose

We calculated the total number of reimbursements of a pain medication and the total amount of substance per claim. We calculated the total amount of substance by calculating the number of pills per reimbursement × strength of the substance. For each pharmaceutical class of pain medications, the total and average numbers of reimbursed pain medication and the cumulative dose in milligrams (mg) of the active pharmaceutical substance were calculated and reported for each year between 2008 and 2018. Wherever possible we calculate the cumulative dose per drug class: paracetamol, metamizole, weak opioids, and strong opioids.

#### Morphine Equivalent Dose (MED)

To account for the different potencies of opioids, the MED was calculated for each opioid (weak and strong) as follows: Strength of opioid drug in mg per unit × quantity of units per reimbursed package × number of packages × conversion factor for morphine equivalents. The equianalgesic dose conversions are only estimates and cannot account for individual variability in genetics and pharmacokinetics. Wherever available we used conversion factors provided by the Swiss Agency for Therapeutic Products (Swissmedic, agency comparable to the US Food and Drug Administration, FDA) or the morphine equivalent conversion factor per mg of opioid was based on the CONSORT classification (CONsortium to Study Opioid Risks and Trends [35]). For more details see Online Appendix 1, Table [31]. The MED calculation for fentanyl patches assumes that one patch delivers the dispensed (and bioavailable) mcg per hour over 72 h. The calculation of the total dose in mg per active substance and then converted it into the total bioavailable MED dose in mg equals. For example, fentanyl patches were calculated as follows: (mcg/h, according to the package reimbursed) × 72 h × number of patches per package × number of packages reimbursed × 100 [fentanyl conversion factor mg_Morphine_/mg_Fentanyl_])/1000. For example, the total MED in mg for one package containing 10 fentanyl patches that each delivers 12mcg per hour is calculated as follows: 12 mcg/h × 72 h × 10 patches × 100 = 864,000 mcg = 864 mg. For transdermal buprenorphine patches the assumption is that one patch delivers the dispensed (and bioavailable) mcg per hour over 96 h. The total MED dose in milligram equals (mcg/h according to the package reimbursed × 96 h × number of patches per package × number of packages reimbursed × 95 [buprenorphine conversion factor])/1000.

#### Treatment Days

Treatment days were calculated using the cumulative dose of substance divided by the DDD. For opioids we calculated in addition to total MED the treatment days as follows: total MED per substance/DDD. Although the DDD for non-opioid medications is useful to calculate the treatment duration, treatment durations in strong opioids are influenced by the strength of the prescribed opioid. Therefore, the treatment days calculated for strong opioids need to be interpreted with caution.

#### Direct Health Care Costs of Pain Medication

Based on the reimbursed pain medications, it was possible to directly calculate the medical costs attributed to pain medication use per case.

### Statistical Analysis

Descriptive statistics included median and interquartile range for the continuous parameters, and percentages for the categorical outcomes. Percentage changes in pain medication use, treatment days, and costs per pain medications was calculated as $\left[ {{{\left( {{\text{Value 2}}0{18} - {\text{Value 2}}00{8}} \right)} \mathord{\left/ {\vphantom {{\left( {{\text{Value 2}}0{18} - {\text{Value 2}}00{8}} \right)} {\left| {{\text{Value 2}}00{8}} \right|}}} \right. \kern-0pt} {\left| {{\text{Value 2}}00{8}} \right|}}} \right]\, \times \,{1}00$. Market shares were calculated as percentages of cases with use of a given pain medication group with respect of all claims with use of at least one pain medication. The difference in market share was calculated as the percentage change between 2018 and 2008. We assessed differences in pain medication use across Swiss Cantons by calculating the proportion of pain medication use per 1000 cases with MSK injuries. We compared variation in pain medication use by calculating the extremal quotient of variation (EQ, the highest divided by the lowest proportion). Statistical analyses were done using SAS statistical analysis software version 9.3 (SAS Institute Inc., Cary, NC, USA).

### Figures and Tables

All figures and tables were created by the authors. We used Microsoft Publisher, Microsoft Excel, R version 4.12 (2021-11-01) and R-Studio 2022.07.0 to create the figures.

## Results

**Fig. 1:**
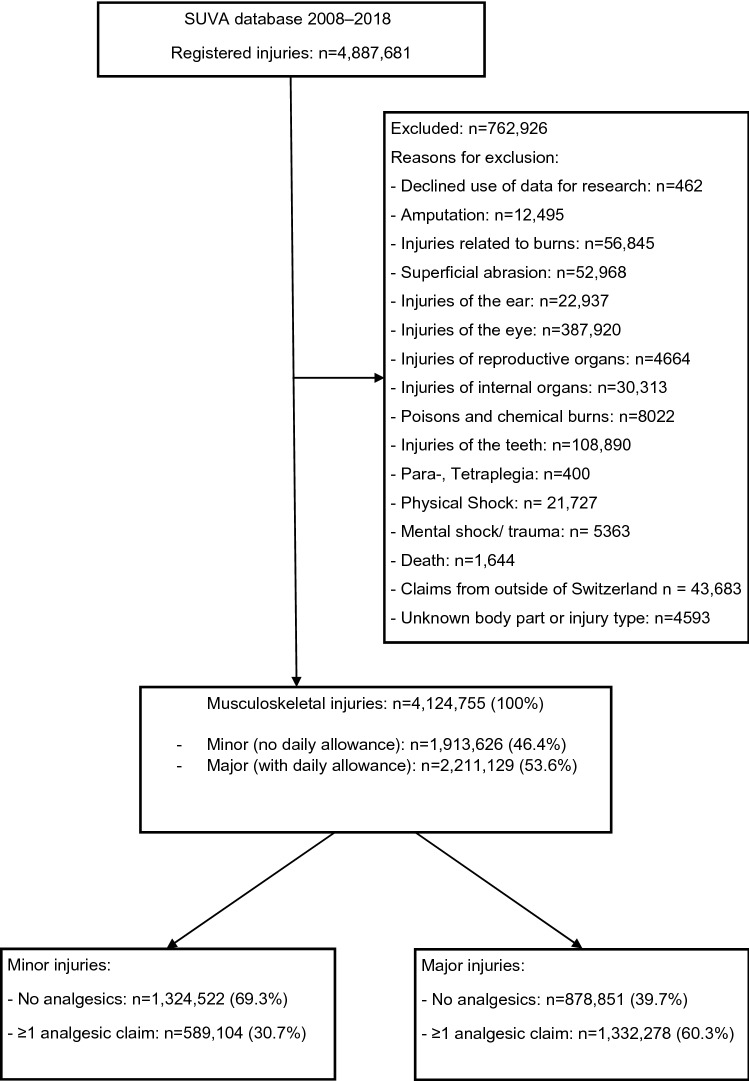
Study flow

Between 2008 and 2018, 4,887,681 injuries were registered (Fig. 1). After exclusion of 762,926 injuries, we analyzed 4,124,755 (minor injuries 1,913,626 (46.4%), major injuries 2,211,129 (53.6%)). Main reasons for exclusion were injuries of the eye and teeth, internal organs, burns and superficial abrasions. In total, 1,921,382 cases with MSK injuries (46.6% of all eligible injuries) had ≥ 1 pain medication(s) reimbursed and were further analyzed. Out of all MSK injuries with ≥ 1 pain medication(s), 589,104 were minor (30.7%) and 1,332,278 (60.3%) major injuries.

### Baseline Characteristics

Most cases were in male (79.4%; Table 1), cases were equally distributed across the three age groups (< 30 years 34.7%, 30 to < 45 years 32.2%, and 45–65 years 33.1%). The average number of treatment days of any pain medication per case was 30.6 daily doses. The total medical expense for pain medication was 58 million Swiss francs with annual costs of 5.3 million Swiss francs. The average expenses for pain medication were 30.06 Swiss francs per person and the medical expenses per daily dose was 0.98 Swiss francs. In most cases NSAIDs (85.0%) and paracetamol (46.3%) were used. Metamizole was used in 14.7%, weak opioids in 10.1%, strong opioids in 2.3%, and coxibs in 1.2% of cases.

**Table 1:** Baseline characteristics for musculoskeletal injuries by pain medication use for the first 730 days after the date of the accident

	Injury with ≥ 1 pain medication	Paracetamol	Metamizole	NSAID	Coxibs	Opioid (weak)	Opioid (strong)
Cases n: (%)	1,921,382 (100)	889,703 (100)	283,077 (100)	1,632,984 (100)	23,776 (100)	194,539 (100)	43,610 (100)
Male: n (%)	1,526,487 (79.4)	704,710 (79.2)	222,471 (78.6)	1,305,735 (80.0)	17,603 (74.0)	155,153 (79.8)	35,675 (81.8)
Age groups							
18 to < 30 years: n (%)	666,397 (34.7)	298,013 (33.5)	84,583 (29.9)	568,458 (34.8)	4076 (17.1)	44,536 (22.9)	12,771 (29.3)
30 to < 45 years: n (%)	618,084 (32.2)	291,322 (32.7)	90,508 (32.0)	530,124 (32.5)	7594 (31.9)	67,462 (34.7)	13,678 (31.4)
45 to 65 years: n (%)	636,901 (33.1)	300,368 (33.8)	107,986 (38.1)	534,402 (32.7)	12,106 (50.9)	82,541 (42.4)	17,161 (39.4)
Total expenses (CHF)*	57,759,797	13,781,635	2,670,406	31,349,719	1,839,853	5,650,660	2,467,523
Average expenses per case (CHF)	30.06	15.49	9.43	19.20	77.38	29.05	56.58
Average expenses per treatment day (CHF)	0.98	0.85	1.29	0.88	1.42	2.00	5.70
MED total: mg	164,269,449	N/A	N/A	N/A	N/A	123,886,641	40,382,808
MED per case: mg	85.5	N/A	N/A	N/A	N/A	636.8	926.0
Treatment days total	58,723,907	16,294,133	2,073,578	35,799,186	1,294,949	2,828,846	433,215
Treatment days per case	30.6	18.3	7.3	21.9	54.5	14.5	9.9

*CHF* Swiss Franc; *MED* medication; *N/A* not applicable

*Medical expenses for pain medications in Swiss Francs (CHF) over the 11-year study period. 1 Swiss Franc equals 0.95 Euro or 1.09 US-Dollar

Whereas the proportion of cases with paracetamol and NSAIDs were similarly distributed across age groups, the proportion of cases with metamizole, weak opioids, strong opioids, and coxibs was higher in the 45–65 age group than in the other age groups (Table 1). The treatment days per case was highest in cases using coxibs (54.5 days) followed by NSAIDs (21.9 days), paracetamol (18.3 days), and strong opioids (14.5 days). Medical expenses per case were highest for coxibs (77.38 Swiss francs) and strong opioids (56.58 Swiss francs).

### Changes in Medication Use and Costs Over Time

The proportion of MSK injuries with one or more pain medication increased from 44.2% (163,183 out of 368,845) of to 46.1% (178,447 out of 387,447 injuries) between 2008 and 2018. Whereas the increase of MSK injuries was 5.0%, the increase in the number of respective cases with pain medication was 9.4% (Table 2), we observed larger increase in cases with metamizole (+ 254%), strong opioids (+ 88.4%), coxibs (+ 85.8%), and paracetamol (+ 28.1%). Metamizole increased from 68.3/1000 injuries in 2008 to 221.4/1000 in 2018 (+ 224%). Strong opioid use increased from 17.5 to 30.1/1000 (+ 72%) and coxibs from 8.7 to 14.8/1000 injuries (+ 70.1%). The use of NSAIDs per 1000 injuries decreased from 857.1 to 843.9 (− 1.5%). The average treatment days per case increased for paracetamol (+ 4.1 days or + 26.8% between 2008 and 2018), metamizole (+ 2.7 days; + 46.2%), NSAIDs (+ 1.2 days; + 5.7%). The average treatment days per case decreased for coxibs (− 8.6 days; − 14.0%), strong opioids (− 3.9 days; − 32.5%), and weak opioids (− 1.2 days; − 8.2%).

**Table 2:** Cases with musculoskeletal injuries by pain medication use and by year of injury registration

Year	2008	2009	2010	2011	2012	2013	2014	2015	2016	2017	2018	% change 2008–2018
Injury with ≥ 1 pain medication	163,183	166,574	174,273	174,044	176,417	178,249	178,507	177,132	175,465	179,091	178,447	+ 9.4
Paracetamol	67,710	72,758	78,226	79,671	81,409	84,214	84,746	84,208	84,473	85,578	86,710	+ 28.1
Rate/1000 injuries	414.9	436.8	448.9	457.8	461.5	472.5	474.8	475.4	481.4	477.9	485.9	+ 17.1
Metamizole	11,152	14,138	17,240	20,177	23,392	25,878	28,607	31,493	33,955	37,544	39,501	+ 254
Rate/1000 injuries	68.3	84.9	98.9	115.9	132.6	145.2	160.3	177.8	193.5	209.6	221.4	+ 224
NSAID*	139,857	142,056	147,895	148,860	149,997	151,503	151,909	150,786	148,761	151,772	150,588	+ 7.7
Rate/1000 injuries	857.1	852.8	848.6	855.3	850.2	850.0	851.0	851.3	847.8	847.5	843.9	− 1.54
Coxibs	1425	1582	2077	2084	2214	2316	2271	2334	2209	2616	2648	+ 85.8
Rate/1000 injuries	8.7	9.5	11.9	12.0	12.6	13.0	12.7	13.2	12.6	14.6	14.8	+ 70.1
Weak opioids	16,062	17,018	18,332	17,671	18,118	18,499	17,767	17,730	17,303	18,243	17,796	+ 10.8
Rate/1000 injuries	98.4	102.2	105.2	101.5	102.7	103.8	99.5	100.1	98.6	101.9	99.7	+ 1.32
Strong opioids	2854	3057	3346	3535	3635	3822	4017	4411	4497	5059	5377	+ 88.4
Rate/1000 injuries	17.5	18.4	19.2	20.3	20.6	21.4	22.5	24.9	25.6	28.3	30.1	+ 72

Medication use for the first 730 days after the date of the accident per year

*NSAID, non-steroidal anti-inflammatory drugs

**Fig. 2:**
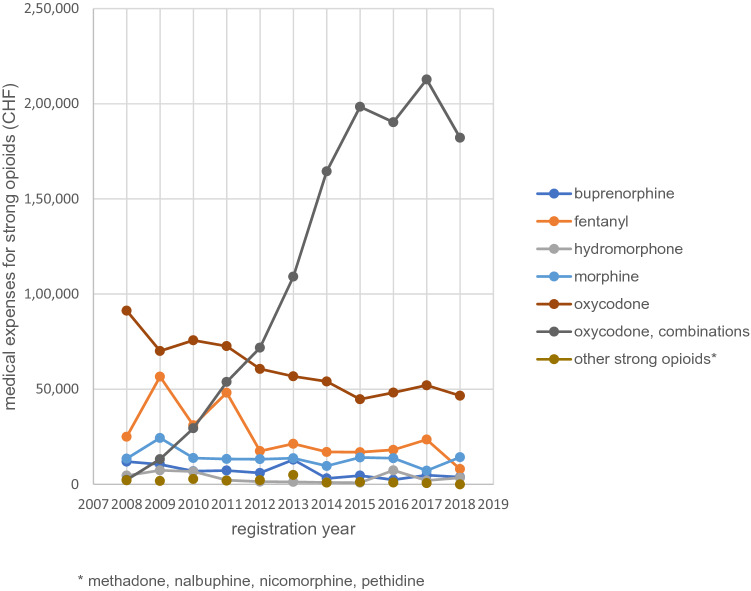
Changes in injuries with pain medications per 1000 injuries with at least one pain medication between 2008 and 2018

The increase in strong opioids was comparable in minor (+ 91.4%) and major injuries (+ 88.3%, Table 3). The increase in metamizole (+ 390.6%) and coxibs (+ 115.5%) was larger in minor injuries compared to major injuries (+ 238.7% and + 80.6%, respectively). The changes in the average treatment days per case was comparable in all pain medications in major and minor injuries. The costs per daily dose decreased in all pain medication except for strong opioids. In strong opioids, an increase of costs per daily dose by 192.4% in minor and 34.0% in major injuries was observed. The increase in costs for strong opioids was mainly due to increased use in oxycodone combinations (Fig. 2).

**Table 3:** Changes in pain medication use in minor and major cases with musculoskeletal injuries and expenses

Year	2008 Cases	2018 Cases	% change 2008–18	Treatment days per case 2008	Treatment days per case 2018	% change 2008–2018	Costs per treatment day 2008	Costs per treatment day 2018	% change 2008–2018
Minor injuries	49,167	51,724	+ 5.2	15.2	18.7	+ 22.8	1.05	0.78	− 25.6
Paracetamol	13,580	17,084	+ 25.8	9.7	13.7	+ 41.2	0.86	0.62	− 27.3
Metamizole	1137	5579	+ 390.6	3.8	5.8	+ 51.1	1.52	1.08	− 28.9
NSAID*	41,503	42,885	+ 3.3	14.0	15.3	+ 9.5	1.05	0.78	− 25.8
Coxibs	213	459	+ 115.5	44.4	41.3	− 6.9	1.47	1.22	− 17.1
Weak opioids	2052	1922	− 6.3	10.0	11.5	+ 14.8	2.07	1.58	− 23.3
Strong opioids	116	222	+ 91.4	6.0	5.1	− 15.7	1.79	5.22	+ 192.4
Major injuries	114,016	126,723	+ 11.1	32.1	38.1	+ 18.8	1.17	0.87	− 25.5
Paracetamol	54,130	69,626	+ 28.6	16.8	20.9	+ 24.6	1.06	0.74	− 29.7
Metamizole	10,015	33,922	+ 238.7	6.0	8.9	+ 48.2	1.67	1.12	− 32.8
NSAID	98,354	107,703	+ 9.5	24.0	25.0	+ 4.0	1.03	0.76	− 26.6
Coxibs	1212	2189	+ 80.6	64.2	55.0	− 14.3	1.54	1.26	− 17.8
Weak opioids	14,010	15,874	+ 13.3	15.8	14.1	− 10.7	2.41	1.63	− 32.6
Strong opioids	2738	5155	+ 88.3	12.1	8.1	− 32.8	4.52	6.06	+ 34.0

Costs per treatment day = costs for pain medications divided by treatment days

^*^NSAID non-steroidal anti-inflammatory drugs

Online Appendix 2 summarizes the percent differences in market share of pain medications between 2008 and 2018. The difference in percent market share was for metamizole + 15.3%, paracetamol + 7.1%, and strong opioids + 1.3% between 2008 and 2018. The market share difference for metamizole and strong opioids was higher in major injuries compared to minor injuries (metamizole + 18.0% vs. + 8.5%, strong opioids + 1.7% vs. + 0.2%) (Online Appendix 3).

### Variation in Pain Medication Use Across Swiss Cantons

**Fig. 3:**
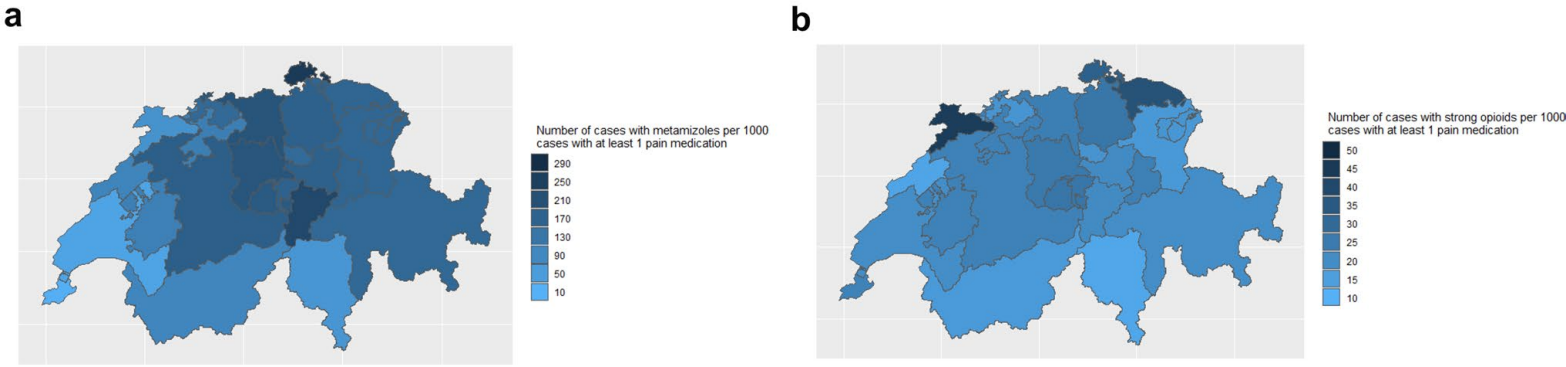
**a** Metamizole use per 1000 cases with musculoskeletal injuries per Swiss canton. **b** Strong opioid use per 1000 cases with musculoskeletal injuries per Swiss canton

The variation across Swiss cantons in pain medication use per 1000 accident cases was very low (EQ of < 2) for paracetamol (EQ 1.9) and NSAIDs (EQ 1.2). The EQ was 2.6 for coxibs, 3.8 for weak opioids, and 3.9 for strong opioids. The largest variation was observed for metamizole (EQ 19.5) with regions in which metamizole was rarely used (e.g., Geneva in 16.1/1000 cases, Vaud in 53.5/1000 cases, Fig. 3a) and very high use (e.g., Schaffhausen in 314.2/1000 cases, Uri 300.2/1000 cases). Higher use in strong opioids were observed in the Cantons Jura (62.0/1000 cases, Fig. 3b), Thurgau (52.7/1000 cases), and Schaffhausen (44.6/1000 cases). Low use in strong opioids was observed in the cantons Ticino (15.8/1000 cases), Neuchatel (18.5/1000 cases), and Valais (21.8/1000 cases) indicating a somewhat lower use in strong opioids in the French and Italian speaking cantons.

## Discussion

In this analysis of more than 4 million MSK injuries between 2008 and 2018, we observed an increase in claims with pain medications. In most injuries NSAIDs were used. Weak opioids, metamizole, and coxibs were used in older adults. Over time, we observed an increase in most pain medications in minor and major injuries. The increase was disproportionally large for metamizole, strong opioids, and coxibs. We observed an equally large increase on strong opioid in minor and major injuries indicating a more liberal prescription practices towards strong opioids also in minor musculoskeletal injuries. This increase was associated with a substantial increase in treatment costs and was mainly due to the increased use in oxycodone combinations. We also observed substantial variation in pain medication use across Swiss cantons especially for metamizole, strong opioids, and weak opioids. French and Italian speaking areas showed a somewhat lower use in strong opioids and metamizole indicating cultural differences between language regions.

### Results in the Light of the Literature

According to analyses using consumer data, Switzerland was among the four top prescriber of opioids worldwide [36]. Between 2010 and 2012, the defined daily dose (DDD) per 100,000 was well above 50,000 in the U.S. followed by 30,000 in Canada. Switzerland had a comparable rate (approximately 20,000 DDD per 100,000 people) to Germany, Austria, and Denmark [37]. In the U.S. and Canada, the high rates were also associated with an opioid crisis with soaring opioid-related morbidity and mortality. To date, no such crisis has been observed in Switzerland despite the very high opioid consumer rates. The main reason is most likely, that consumer data also include opioid use within drug substitution programs covered by health insurers in Switzerland. Other factors such as improved palliative care and access to opioids in patients requiring strong pain medications may explain an increased use of opioids also observed across other European countries. The consequences of an increased use of opioids in Europe may be less obvious compared to the opioid crisis observed in North America [28, 38]. However, there is some evidence that an increase in opioid use resulted in an increase in mortality from opioid poisoning in the Netherlands [39] and opioid poisonings increased in Switzerland between 2000 and 2019 by 177% [40]. Thus, an increased opioid use observed on a population level in many European countries may have potential wide-reaching consequences and are of great importance on the individual and societal level. The underlying reasons are not well understood, and a better understanding may help to address inappropriate or ineffective pain management. Switzerland, a country with universal health care coverage, offers a unique opportunity to assess potential underlying factors also relevant for other countries.

The overall pain medication uses in patients presenting with new MSK conditions to the primary care physician in the U.K. were comparable to our study [41]. The current study showed in a relatively homogenous population of MSK injuries a large increase in strong opioid use clearly indicating that the reluctance of opioid use in minor injuries decreased. In MSK injuries, the use of opioids is not recommended because of the side effects and the very small effect on pain and function [18, 27]. Opioid use after an injury was associated with lower recovery rates and return to work in observational studies [42–46]. Early opioid use after an injury was associated with a higher rate of surgery [47], longer time to return to work [48], and higher rate of long-term disability benefits [49]. In acute occupational low back pain, opioid use within the first 15 days was associated with longer disability duration compared to no opioid use [45]. Further, higher opioid dose such as > 450 mg morphine equivalent (MEQ) was associated with an average of 69 days longer disability [45]. Although higher initial dose of opioids in workers with back injuries was associated with an increased risk of long-term opioid use [50], this finding may also be due to injury severity or pain intensity. In chronic MSK pain, long-term opioid resulted in a poorer quality of life without improvement in function or pain control [19, 25, 51]. Opioid dose reduction or discontinuation may lead to a reduction of pain severity, improved function, and life quality in patients with long-term opioid treatment [26]. Thus, increased opioid use in minor MSK injuries may have unintended consequences on recovery after the injury and be of great long-term consequences from an individual and a societal perspective.

We observed a large regional variation of opioid and metamizole use in MSK injuries. Such differences in geographically close regions may be explained by cultural differences in the use of pain medication on a prescriber and patient level. Factors associated with variation in preference sensitive surgical procedures in Switzerland included physician preferences, cultural differences, socioeconomic factors, and health literacy [52–55]. In the neighboring country Germany, a systematic review of opioid data from different data sources showed a variation in strong opioid use comparable to our study (EQ 3.5; 87.0 DDD/100 insured persons to 304.8 DDD/100 insured persons) [56]. The regional prevalence of opioid use ranged in the German federal states from 1.13% (Baden-Württemberg) to 1.67% (Lower Saxony). Equally large variations were observed across Northern, Eastern, and Southern England (EQ 3.8, Manchester 53.1 DDD/1000 registrants per day, Newcastle 48.9, Birmingham 35.3, and London 13.9 DDD/1000 registrants per day) with more opioid use being associated with greater deprivation at a population level [57]. Whether socioeconomic factors may also explain regional differences in pain medication use between Swiss cantons is unknown. Wide variations not only in opioid use but also in metamizole use observed in this study, but also across Germany [58], indicate physician preferences may play an important role. Thus, the findings warrant further studies on underlying reasons and to assess potential interventions on a patient and prescriber level. To prevent potential unintended consequences of low value care such as opioid use in minor MSK injuries, further studies should assess factors on a prescriber and patient level that result in practice changes. Several state-level policy interventions in Washington State, U.S.A., has been shown to improve safe prescribing which should prevent long-term opioid use and reduce opioid-related deaths in injured workers [59, 60]. Whether such interventions are equally effective in European countries is unknow [61].

### Strengths and Limitations

Although the Suva database provides a comprehensive insight into medication prescription practices of injured workers in Switzerland, there are several limitations that need to be discussed. First, we have no clinical information on the severity and type of injury. Second, MSK injuries were based on claim-reports and may not align with medical reports and physician’s diagnosis. Third, although we had information on the number of pain medication that were prescribed, we had no information on whether patients in fact did take them or not. Individual pain medication use varies widely. Further, we were not able to assess over-the-counter pain medication use. Although some patients may have purchased pain medication over the counter, the insurance covers all costs and thus, we expect that patients will rather fill prescriptions than pay out of pocket costs for medications used to treat their injury. However, remaining medication such as pills and patches may be used at a later stage and may also result in unintended consequences [62].

### Implication for Practice

Despite guideline recommendations to only use strong opioids in severely injured cases and cases with contraindications for other medications, opioids are increasingly used also in minor MSK injuries. Physicians should be aware of potentially unintended effects of early use of strong opioids and restrict opioid use to selected patients. Further, unused pills may be a source for overdose or for other reasons with potentially severe health consequences [62].

Initiatives to reduce frequency of new prescription of opioids in MSK disorders appear effective at least in some jurisdictions [63]. The reasons for the reduction seem to be multifactorial. Such factors likely include increased awareness of prescribers and patients, drug monitoring programs, adapted remuneration systems, opioid education, and access to behavioral health services [64–66]. However, it is still unclear which interventions are the most effective without enforcing threshold for prescribing [67, 68].

### Implication for Research

Future studies should assess the long-term impact of increased opioid and pain medication use in minor MSK injuries observed in the current study. Moreover, more research is needed to assess factors that may explain variation in care across regions such as access to care, socioeconomic factors, health literacy, and physicians` attitude towards the efficacy of pain medications and proficiency in pain management. Studies should also assess the efficacy of interventions on a policy level to improve safe prescribing and care in MSK injuries.

## Conclusion

We observed a disproportionate increase in metamizole, strong opioids, coxibs, and paracetamol prescriptions even in minor musculoskeletal injuries between 2008 and 2018. Whereas treatment costs decreased for all pain medications, there was a substantial increase in strong opioids. A more liberal prescription practice of opioids conflicts with current evidence-based practice recommendations and need to be addressed by physicians and policy makers. The use of strong opioids in minor injuries not requiring opioids may have substantial consequences for the individual and society.

### Supplementary Information

Below is the link to the electronic supplementary material.

Supplementary file1 (PDF 362 kb)

Supplementary file2 (JPG 167 kb)

Supplementary file3 (JPG 256 kb)

Supplementary file4 (JPG 41 kb)
Fig. 3c Paracetamol use per 1000 cases with musculoskeletal injuries per Swiss canton.

Supplementary file5 (JPG 36 kb)
Fig. 3d NSAID use per 1000 cases with musculoskeletal injuries per Swiss canton.

Supplementary file6 (JPG 38 kb)
Fig. 3e Coxibs use per 1000 cases with musculoskeletal injuries per Swiss canton.

Supplementary file7 (JPG 41 kb)
Fig. 3f Weak opioid use per 1000 cases with musculoskeletal injuries per Swiss canton

## Data Availability

The datasets generated during and/or analyzed during the current study are available from the corresponding author on reasonable request.
