# Supplementary material for: Increased Use and Large Variation in Strong Opioids and Metamizole (Dipyrone) for Minor and Major Musculoskeletal Injuries Between 2008 and 2018: An Analysis of a Representative Sample of Swiss Workers
Source: J Occup Rehabil. 2023 Apr 11;34(1):157–68. doi: 10.1007/s10926-023-10115-5 (PMC10899285; doi:10.1007/s10926-023-10115-5)
Supplement: Supplementary file 1 — Supplementary file1 (PDF 362 kb) [file 10926_2023_10115_MOESM1_ESM.pdf]

Weak opioids

ATC code	Adm.R	DDD	U	Morpheq	DDD mg	Long / Short	Substance
N02AA08	O	0.15	g	0.1	150	Long	Dihydrocodeine
N02AA59	O	120	Mg*	0.15	120	Short	Codeine, combinations
N02AA59	R	80	Mg*	0.15	80	Short	Codeine, combinations
N02AX01	O	0.2	G	0.2	200	Short	Tilidine
N02AX02	O	0.3	G	0.2	300	Short	Tramadol
N02AX02	O	0.3	G	0.2	300	Long	Tramadol
N02AX02	P	0.3	G	0.2	300	Short	Tramadol
N02AX02	R	0.3	G	0.2	300	Short	Tramadol
N02AX06	O	0.4	G	0.4	400	Short	Tapentadol
N02AX06	O	0.4	G	0.4	400	Long	Tapentadol
N02AX52	O	150	mg	0.2	150	Short	Tramadol combinations
N02AJ13	O	0.3	G	0.2	300	Short	Tramadol and paracetamol
N02AJ06	O	0.12	G	0.15	120	Short	Codeine and paracetamol
N02AJ06	R	0.12	G	0.15	120	Short	Codeine and paracetamol

Strong opioids

ATC code	Adm.R	DDD	U	Morpheq	DDD mg	Long / Short	Substance
N02AA01	O	0.1	g	1	100	Short	Morphine
N02AA01	O	0.1	g	1	100	Long	Morphine
N02AA01	P	30	mg	1	30	Short	Morphine
N02AA01	R	30	mg	1	30	Short	Morphine
N02AA03	O	20	mg	5	20	Short	Hydromorphone
N02AA03	O	20	mg	5	20	Long	Hydromorphone
N02AA03	P	4	mg	17.5	4	Short	Hydromorphone
N02AA04	O	30	mg	1	30	Short	Nicomorphine
N02AA04	P	30	mg	3	30	Short	Nicomorphine
N02AA04	R	30	mg	1	30	Short	Nicomorphine
N02AA05	O	75	mg	1.5	75	Short	Oxycodone
N02AA05	O	75	mg	1.5	75	Long	Oxycodone

N02AA05	P	30	mg	3	30	Short	Oxycodone
N02AA55	O	75	mg	1.5	75	Long	Oxycodone, combinations
N02AB02	P	0.4	G	0.4	400	Short	Pethidine
N02AB03	N	0.6	mg	100	0.6	Short	Fentanyl
N02AB03	SL	0.6	mg	100	0.6	Short	Fentanyl
N02AB03	TD	1.2	mg	112.5	1.2	Long	Fentanyl
N02AE01	P	1.2	mg	100	1.2	Long	Buprenorphine
N02AE01	SL	1.2	mg	75	1.2	Long	Buprenorphine
N02AE01	TD	1.2	mg	91.67	1.2	Long	Buprenorphine
N02AF02	P	80	mg	3	80	Short	Nalbuphine
N07BC01	SL	8	mg	75	8	Long	Buprenorphine
N07BC02	O	25	mg	4.7	25	Long	Methadone
N07BC02	P	25	mg	13.5	25	Short	Methadone
N07BC02	R	30	mg	4.7	30	Short	Methadone

Adm.R, administration route; O, oral; P, parenteral; R, rectal; SL, sublingual; TD, transdermal; N, nasal; DDD, defined daily dose is the assumed average maintenance dose per day for a drug used for its main indication in adults [69]; U, unit; morpheq, Morphine Equivalent Conversion Factor (strength of opioid drug in mg per unit x quantity of units per reimbursed package x number of packages x conversion factor for morphine equivalents. Transmucosal fentanyl conversion MED in milligram for transdermal fentanyl patches were calculated: (mcg/hour (according to the package reimbursed) x 72 hours’ x number of patches per package x number of packages reimbursed x 100 [fentanyl conversion factor]) / 1000. MED in milligram for transdermal buprenorphine patches were calculated: (mcg/h according to the package reimbursed x 96 hours’ x number of patches per package x number of packages reimbursed x 95 [buprenorphine conversion factor]) / 1000.

*All DDD are based on the WHO ATC provided daily dose except for codeine. In Switzerland, codeine is available in combination with paracetamol for pain treatment. No DDD from the WHO were available for codeine-combinations. Therefore, the average treatment dose of the combinations was used to calculate DDD: e.g. Co-Dafalgan® four times daily = 4x20mg codeine.
