# Supplementary figures and images for: Increased Use and Large Variation in Strong Opioids and Metamizole (Dipyrone) for Minor and Major Musculoskeletal Injuries Between 2008 and 2018: An Analysis of a Representative Sample of Swiss Workers
Source: J Occup Rehabil. 2023 Apr 11;34(1):157–68. doi: 10.1007/s10926-023-10115-5 (PMC10899285; doi:10.1007/s10926-023-10115-5)

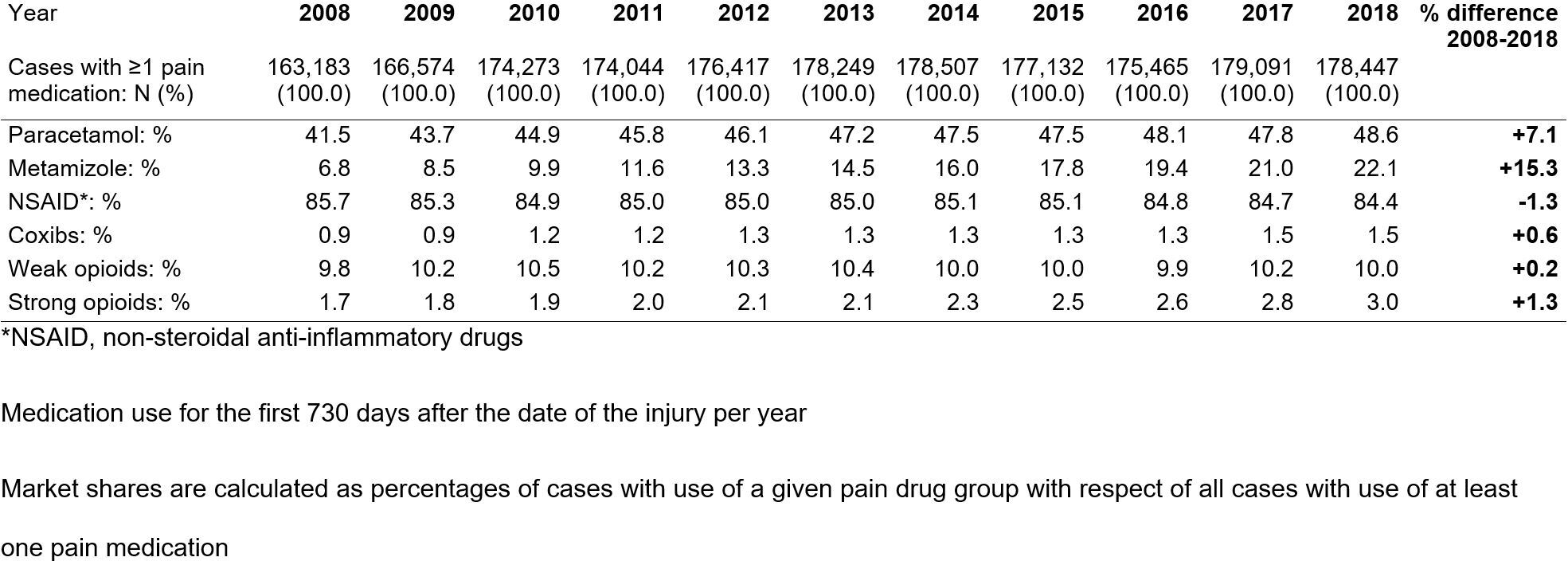

Supplement: Supplementary file 2 — Supplementary file2 (JPG 167 kb) [file 10926_2023_10115_MOESM2_ESM.jpg]

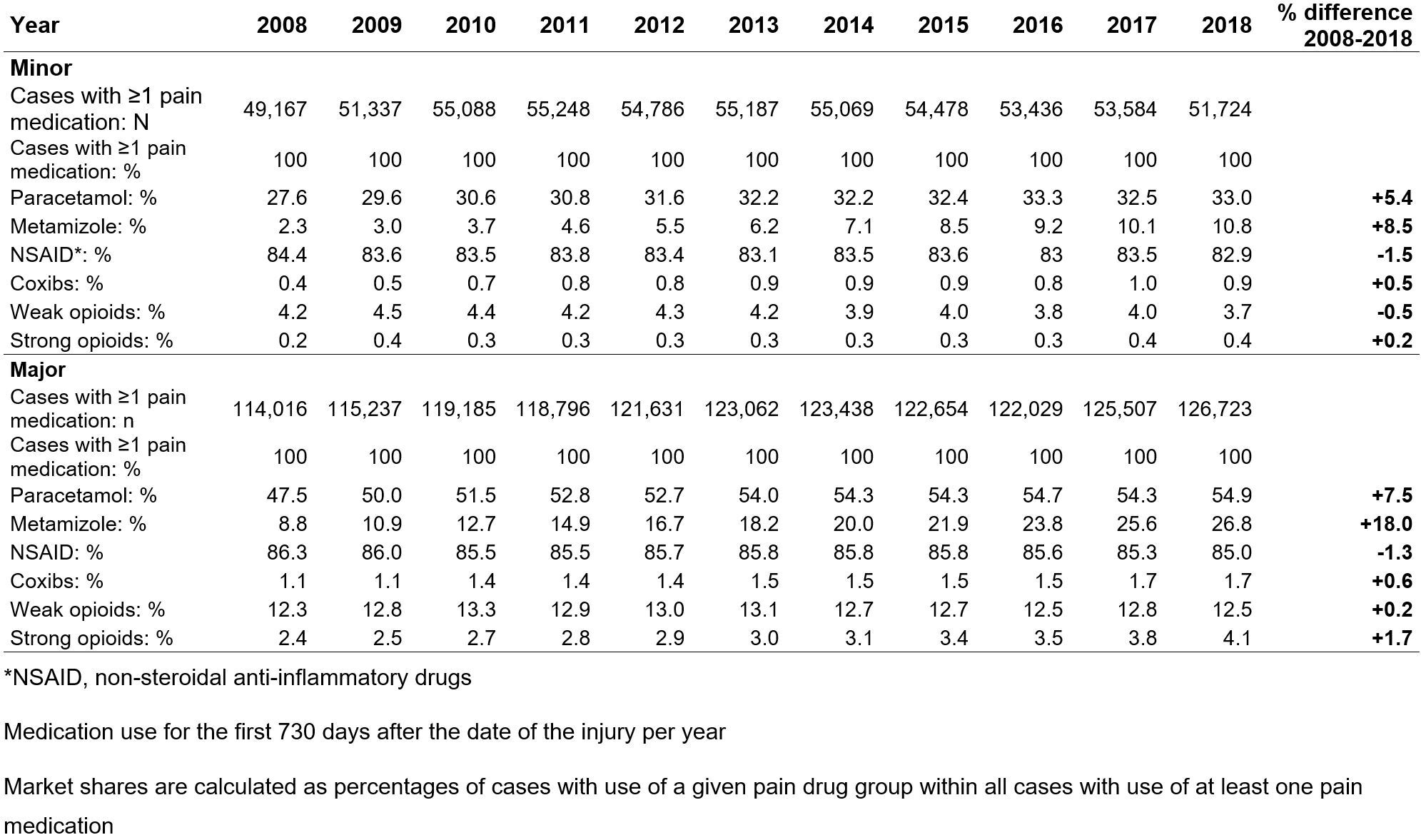

Supplement: Supplementary file 3 — Supplementary file3 (JPG 256 kb) [file 10926_2023_10115_MOESM3_ESM.jpg]

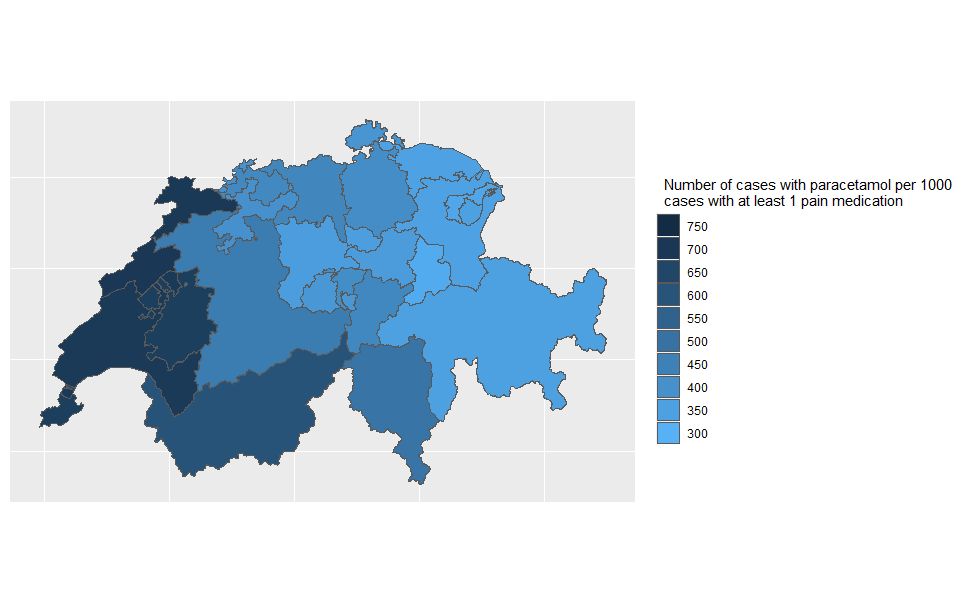

Supplement: Supplementary file 4 — Supplementary file4 (JPG 41 kb) Fig. 3c Paracetamol use per 1000 cases with musculoskeletal injuries per Swiss canton. [file 10926_2023_10115_MOESM4_ESM.jpg]

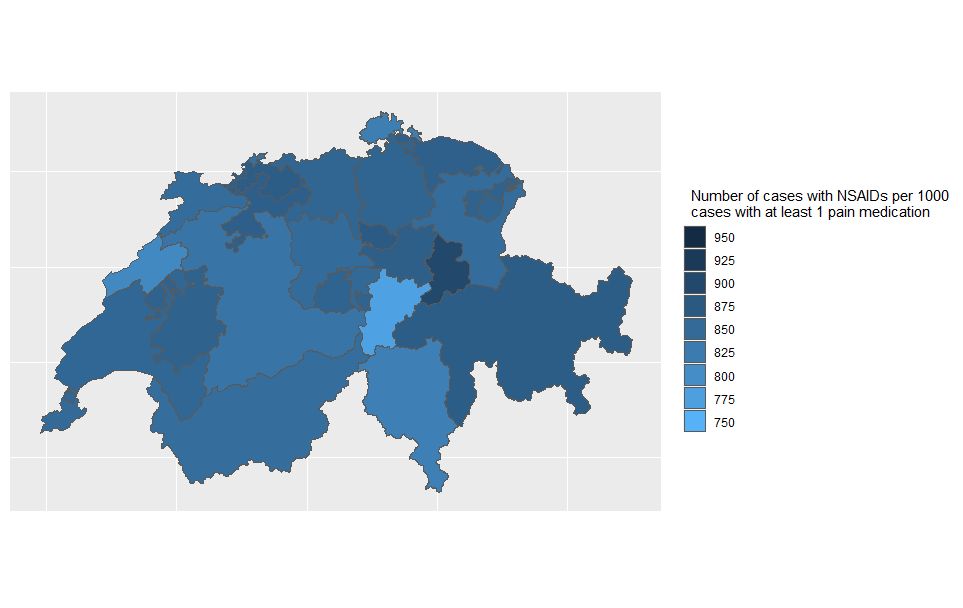

Supplement: Supplementary file 5 — Supplementary file5 (JPG 36 kb) Fig. 3d NSAID use per 1000 cases with musculoskeletal injuries per Swiss canton. [file 10926_2023_10115_MOESM5_ESM.jpg]

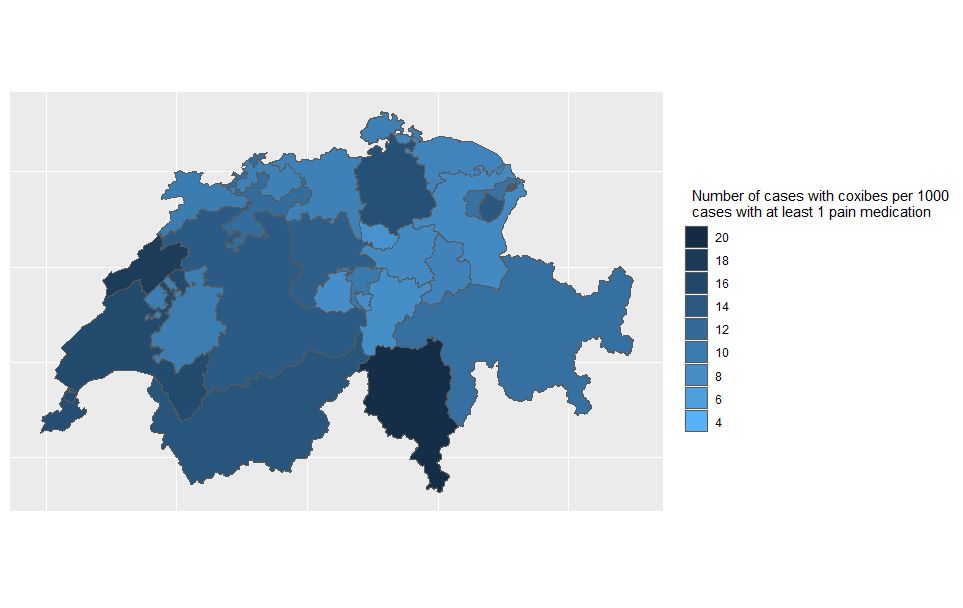

Supplement: Supplementary file 6 — Supplementary file6 (JPG 38 kb) Fig. 3e Coxibs use per 1000 cases with musculoskeletal injuries per Swiss canton. [file 10926_2023_10115_MOESM6_ESM.jpg]

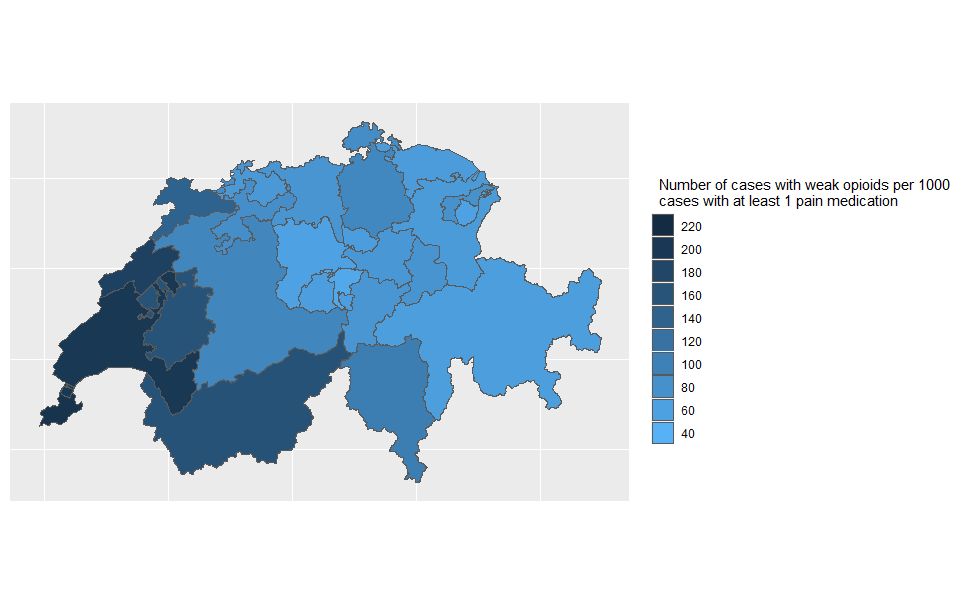

Supplement: Supplementary file 7 — Supplementary file7 (JPG 41 kb) Fig. 3f Weak opioid use per 1000 cases with musculoskeletal injuries per Swiss canton [file 10926_2023_10115_MOESM7_ESM.jpg]
